# Zebrafish tumour xenograft models: a prognostic approach to epithelial ovarian cancer

**DOI:** 10.1038/s41698-024-00550-9

**Published:** 2024-02-27

**Authors:** Gabriel Lindahl, Sebastian Fjellander, Karthik Selvaraj, Malin Vildeval, Zaheer Ali, Rusul Almter, Anna Erkstam, Gabriela Vazquez Rodriguez, Annelie Abrahamsson, Åsa Rydmark Kersley, Anna Fahlgren, Preben Kjølhede, Stig Linder, Charlotta Dabrosin, Lasse Jensen

**Affiliations:** 1 Department of Oncology and Department of Biomedical and Clinical Sciences, Linköping University, Linköping, Sweden; 2 BioReperia AB, Linköping, Sweden; 3 Linköping University, Department of Health, Medicine and Care, Division of Diagnostics and Specialist Medicine, Linköping, Sweden; 4 Linköping University, Department of Biomedical and Clinical Sciences, Linköping, Sweden; 5 Department of Obstetrics and Gynecology and Department of Biomedical and Clinical Sciences, Linköping University, Linköping, Sweden

**Keywords:** Cancer models, Prognostic markers, Translational research

## Abstract

Epithelial ovarian cancer (EOC) is the gynaecological malignancy with highest mortality. Although adjuvant treatment with carboplatin and paclitaxel leads to an objective response in ~80% of these patients, a majority will relapse within two years. Better methods for assessing long-term treatment outcomes are needed. To address this, we established safe and efficacious doses of carboplatin and paclitaxel using IGROV-1 zebrafish-CDX models. Then fluorescently-labelled cell suspensions from 83 tumour biopsies collected at exploratory laparotomy of women with suspected EOC were generated and 37 (45%) were successfully implanted in zebrafish larvae. Among these 19 of 27 pathology-confirmed EOC samples (70%) engrafted. These zebrafish patient-derived tumour xenograft (ZTX) models were treated with carboplatin or paclitaxel and tumour growth/regression and metastatic dissemination were recorded. In a subgroup of nine patients, four ZTX models regressed during carboplatin treatment. All four corresponding patients had >24 months PFS. Furthermore, both ZTX models established from two patients having <24 months PFS failed to regress during carboplatin treatment. Seven of eight models seeding <6 metastatic cells were established from patients having >24 months PFS. In eleven of fourteen patients, FIGO stage I + II or III tumours gave rise to ZTX models seeding <4 or >4 metastatic cells, respectively. In conclusion, ZTX models predicted patients having >24 or <24 months PFS, based on response/no response to carboplatin. Furthermore, high metastatic dissemination in ZTX models correlated to shorter PFS and more advanced disease at diagnosis. These preliminary results suggest that ZTX models could become a useful prognostic tool in EOC treatment planning.

## Introduction

Epithelial ovarian cancer (EOC), the seventh most common female malignancy, accounts for over 150 000 deaths annually worldwide^1^. Approximately 75% of all patients are diagnosed with late-stage disease. Although 70–80% of the patients initially respond to primary therapy, the prognosis is dismal, with a 5-year overall survival for stage III and IV of <40%^1–3^. Hence, for a majority of patients with advanced EOC, the main concern is not the lack of initial response to treatment but the high risk of relapse and acquired drug resistance. Moreover, the patients often suffer from symptoms of progressive disease and accumulated side effects from several lines of treatment. Hence, it is of high priority to identify and select the treatment most likely to provide long-term disease control for each patient.

During the last decade, there has been an advance in next-generation sequencing, which enables rapid and affordable genomic tumour mutation profiling. This development has led to the concept of precision medicine—i.e., tailoring treatment for each patient by looking at the mutation profile and prescribing targeted therapies directed at these mutations^4^. In spite of these advances, conventional non-targeted chemotherapy has, in accordance with clinical guidelines, continued to be the backbone of cancer treatment. The failure of current precision medicine approaches, at least in part, can be explained by the need for specific molecular targets expressed in the patients’ tumours^5^. Moreover, previous clinical trials that identify potential patients eligible for treatment with targeted therapies show that only ~10–40% of the patients have actionable genomic alterations and that less than 10% are actually enrolled in the trials^6,7^. Although the field of precision medicine is rapidly expanding, the possibility of acting on molecular characterization for an individual patient is still rather limited.

As an alternative or complement to genetics and other molecular precision medicine techniques, extensive efforts to develop clinically useful and functional tools for chemotherapy response prediction and treatment planning have recently been evaluated^8,9^. These efforts have resulted in several functional patient-derived tumour models currently being translated from pre-clinical to clinical applications^8^. Among these, in vitro chemotherapy sensitivity and resistance assays (CSRAs)^10^, including both 2D and 3D cultures of patient tumour cells, have been used to test the efficacy of chemotherapeutic agents directly on tumour cells that have been established and growing as 3D organoids in the lab^10^. Several retrospective studies using CSRAs have demonstrated the accuracy of such systems in predicting the outcome of the primary treatment, and a few prospective trials have shown trends towards improved progression-free survival (PFS) in the recurrent disease setting when CSRAs have been used for treatment planning^11^. However, previously evaluated CSRAs have suffered from long assay times (weeks to months), poor technical performance leading to assay failure for a large proportion of the patients, and insufficient validation^12,13^. For patients with EOC, the establishment of a CSRA to evaluate functional treatment outcomes has only been achieved for 10–20% of the patients^14^. Therefore, validated and faster functional assays with better technical performance are needed to support effective treatment planning for EOC patients.

In 2005, Lee et al. successfully xenotransplanted a human malignant melanoma cell line into zebrafish larvae, an accomplishment that has led to the rapid development of zebrafish as a cancer model during the last decade^15–17^. Recently, several proof-of-concept studies have successfully engrafted patient-derived tumour tissues in zebrafish (ZTX) with gastrointestinal^18–20^, haematological^21,22^, urinary bladder^23^, breast^24^, and prostate cancer^25^ cells. In addition, ZTX models have been used to test and predict drug response to both standard treatment as well as novel drug candidates^17,26^. Our research team has recently generated ZTX models from non-small cell lung cancer patients and used these to predict outcomes of first-line treatment as well as the risk of lymph node or metastatic dissemination with >90% accuracy^27^. Importantly, such models were generated from >83% of the patients, and results were available within 5 days of tissue collection (biopsy or surgery), both dramatic improvements compared to the technical performance of CSRAs. Although ZTX models have been shown to have high value within (companion) diagnostics, their potential use for prognostication (i.e., to predict time-to-relapse/progression) has not been studied. Similarly, ZTX models have not previously been applied to EOC, and the accuracy of ZTX models to predict treatment outcomes within this patient group is not known.

Here, we describe a technique that allows for establishing ZTX models from patients with suspected or confirmed EOC with implantation and engraftment success rates of 45% and 70%, respectively. In an explorative sub-cohort, we show that for all four cases of carboplatin sensitivity in ZTX models the corresponding patients exhibited >24 months of PFS. Similarly, for 2 patients with <24 months PFS on adjuvant treatment, the corresponding ZTX models did in both cases also not respond to carboplatin treatment. In addition, we show that low metastatic behaviour in the ZTX models was predictive of >24 months of PFS in 7 of 8 patients and could separate low-grade (I/II) from high grade (III) cancer in 11 of 14 patients. As such, in this translational study, we provide a proof-of-principle of the power of ZTX models for developing prognostic information for EOC. After validation in larger clinical studies, ZTX models could become an important functional precision medicine tool that is relevant for clinical treatment planning.

## Results

### Exposure of zebrafish ovarian cancer models to carboplatin or paclitaxel

**Fig. 1:**
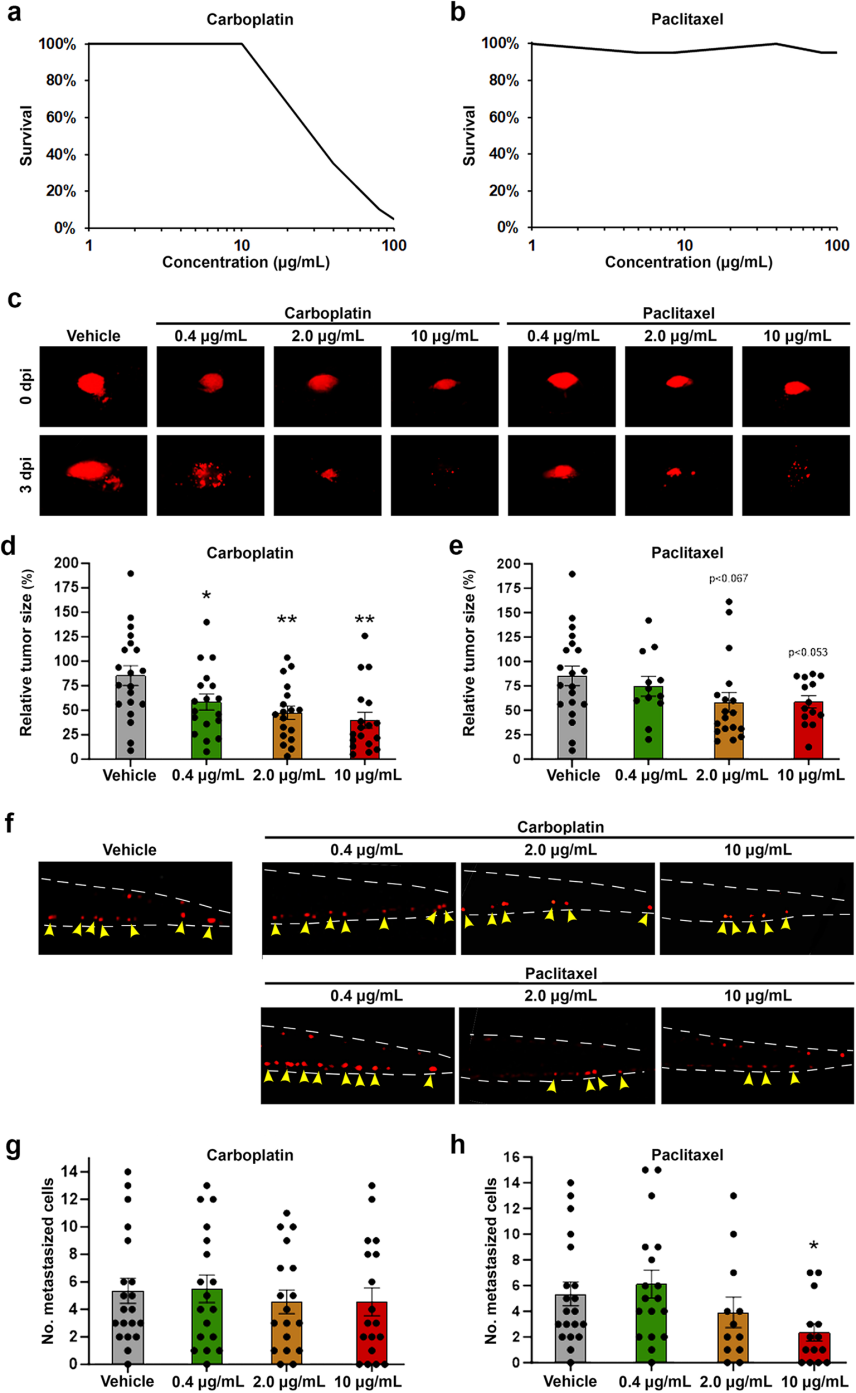
Carboplatin and paclitaxel demonstrate concentration-dependent safety and anti-cancer efficacy in IGROV-1 EOC models. **a**, **b** Curves showing the proportion of embryos surviving treatment with the indicated concentrations of carboplatin (**a**) or paclitaxel (**b**) for three days at 36 °C between 2- and 5-days post fertilization. *N* = 20 embryos per group in three technical replicates. **c** Representative fluorescent micrographs showing the primary implantation site after fluorescently-labelled IGROV-1 tumour cells (red) were implanted into 2-day-old zebrafish larvae, treated with 0.4–10 µM carboplatin or paclitaxel at 36 °C and imaged at 0 days post implantation (dpi) or 3 dpi. **d**, **e** Quantification of relative tumour sizes of IGROV-1 tumour-bearing larvae treated with carboplatin (**d**) or paclitaxel (**e**) from the experiment shown in (**c**). *n* = 12–20 embryos per group in three technical replicates. *p < 0.05, **p < 0.01. **f** Representative fluorescent micrographs showing fluorescently-labelled IGROV-1 tumour cells (red) in the metastatic site in the caudal hematopoietic plexus of 5-day-old zebrafish larvae treated with 0.4–10 µM carboplatin or paclitaxel at 36 °C and imaged at 3 dpi. **g**, **h** Quantification of the number of metastasized IGROV-1 cells after treatment with carboplatin (**g**) or paclitaxel (**h**) from the experiment shown in (**f**). *n* = 12–20 embryos per group in three technical replicates. *p < 0.05.

Carboplatin and paclitaxel, two commonly used drugs for the treatment of patients with EOC, show dramatic differences with regard to their water solubility. Because carboplatin is soluble in water at 10 mg/mL, it can be used for the treatment of zebrafish embryos in aquatic cultures. Carboplatin in concentrations of 40 μg/mL were found to lead to significant embryo mortality after 3 days of incubation at 36 °C, but carboplatin in concentrations of 10 μg/mL did not affect embryo survival or result in any non-lethal toxic phenotype (Fig. 1a). In contrast, paclitaxel has very poor solubility in water (<0.1 μg/mL)^28^. The addition of paclitaxel up to 100 μg/mL did not affect embryo viability, and was not associated with obvious toxicity, likely due to limited solubility and therefore actual exposure (Fig. 1a, b).

We established xenografts from IGROV-1 cells, a commonly used EOC cell line, and treated these with different doses of carboplatin and paclitaxel for 3 days. Carboplatin significantly reduced the tumour volume in a concentration-dependent manner. The highest concentration (10 μg/mL) induced a >50% reduction in tumour volume and was chosen for further studies (Fig. 1c, d). Paclitaxel did not reduce the tumour volume with the same potency as carboplatin, but treatment with the highest concentration (10 μg/mL) caused a regression of the tumours that was borderline significant (*p* < 0.052) (Fig. 1c, e). Unexpectedly, the effects of the two drugs on dissemination of IGROV-1 cells to the primary metastatic site at the caudal hematopoietic tissue differed from their effects on tumour growth. Dissemination was not affected by any of the carboplatin doses tested but was significantly reduced after treatment with 10 μg/mL of paclitaxel (Fig. 1f–h). The finding of dose-dependent effects of paclitaxel on tumour growth and dissemination above the limit of solubility may be explained by dynamic flow from the insoluble phase into water (see Discussion). Therefore, dosing of paclitaxel is difficult to determine. As a result, we used a concentration of 20 μg/mL for further studies, a concentration well below the tolerated concentration (i.e., 100 μg/mL).

### Establishment of ZTX models from cryopreserved tissues

**Fig. 2:**
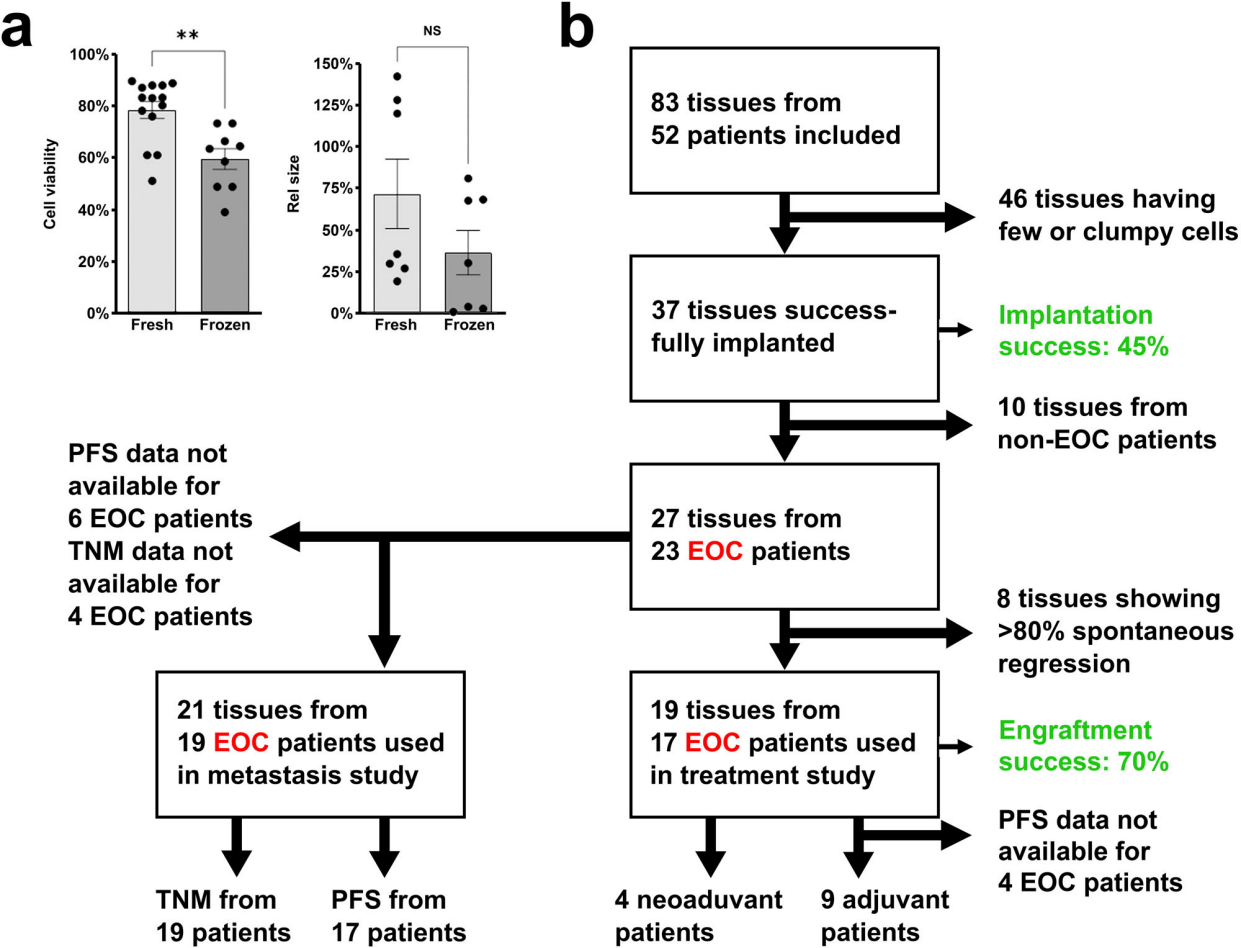
Inclusion and exclusion criteria and handling of patient samples in the clinical study. **a** Quantifications of changes in cell viability and tumour size (rel. size) of cells and xenografts, respectively, generated from either fresh or cryopreserved (frozen) tissue samples. *n* is shown in each graph. NS: non-significant, **p < 0.01. **b** Flow diagram depicting the number of patients included/excluded for the different sub-studies. All initially included patients had suspected EOC based on radiological images. EOC: Epithelial ovarian cancer; PFS: Progression-free survival; TNM: Tumour-Node-Metastasis.

To prepare for ZTX studies based on clinical tumour material, we first examined whether ZTX models could be established from cryopreserved samples of ovarian tumours as this would both provide greater logistical flexibility and facilitate testing of different compounds on the same tumours over time. While a similar number of cells could be extracted from either fresh or cryopreserved tissues, cell viability was slightly lower using cryopreserved samples (Fig. 2a). This decrease, however, did not have practical importance as we found that the median tumour regression observed when implanting cells isolated from cryopreserved tissues was not significantly exceeding that from ZTX models generated using fresh tissues (Fig. 2a). Based on these findings, we continued our examination using cryopreserved samples where all tissues were collected and cryopreserved within 2 h of surgery.

### Characterization of ZTX models generated from EOC patient samples

**Fig. 3:**
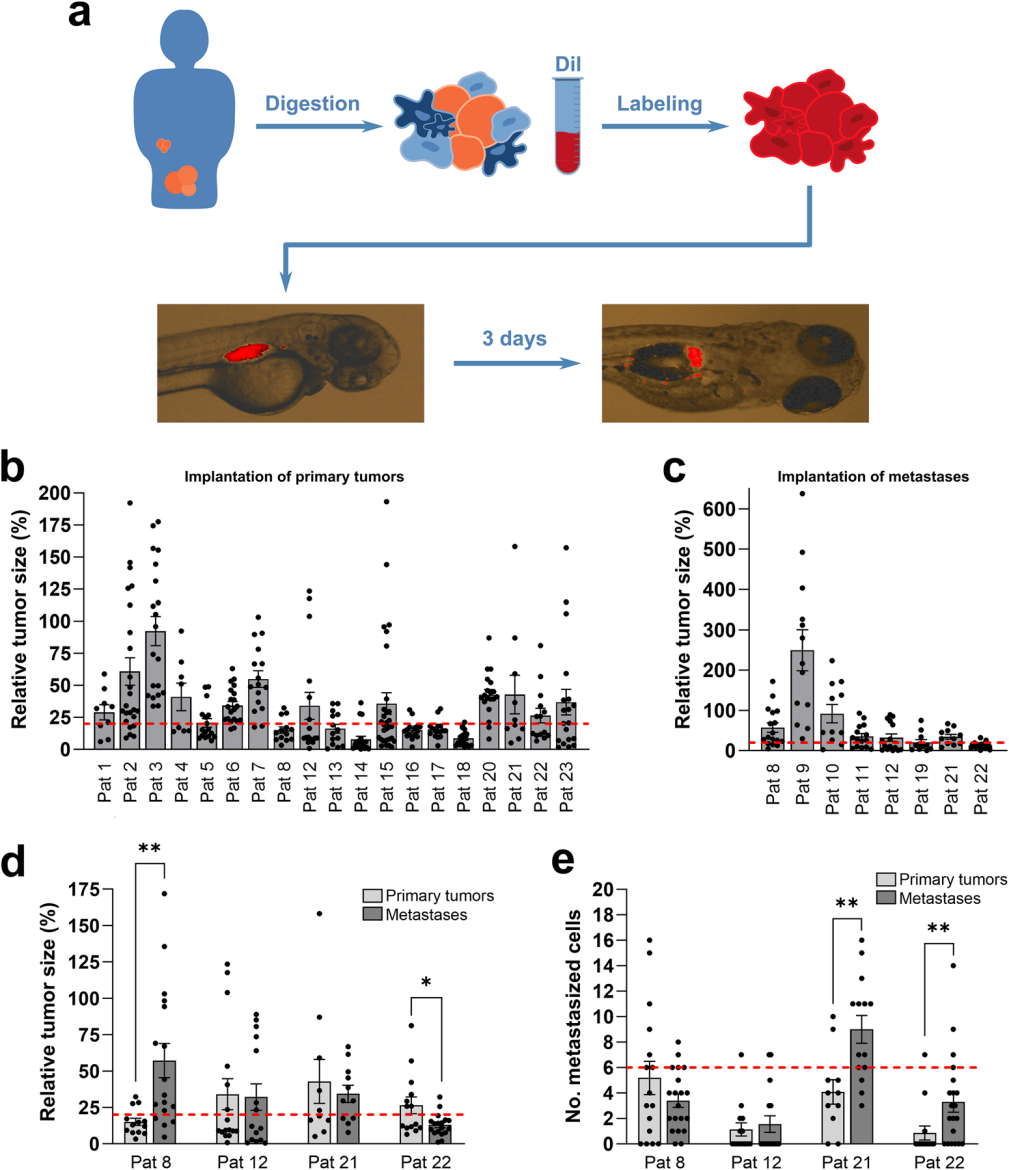
Implantation of EOC ZTX models in zebrafish larvae. **a** Illustration of digestion, staining, implantation, and visualization of EOC tumour samples from primary tumour and metastases transplanted to zebrafish larvae. **b** Quantification of changes in tumour size (rel. size) between day zero and day three for 19 samples from the primary tumour in 19 patients. Cut-off for included models in further studies was those that decreased less than 80% in size (red dotted line). *n* is shown as individual dots in the graph. **c** Quantification of changes in tumour size (rel. size) between day zero and day three for 8 samples from metastatic lesions in 8 patients. Cut-off for including models in further studies were a decrease of less than 80% in size (red dotted line). *n* is shown as individual dots in the graphs. The experiment was done once. **d** Comparison of changes in tumour size (rel. size) between samples from primary tumour and samples from metastases transplanted to the zebrafish larvae. The cut-off of less than 80% regression of the tumour size is shown with a red dotted line. *p < 0.05, **p < 0.01. **e** Comparison of number of cells disseminated to the caudal venous plexus of the zebrafish larvae between samples from primary tumours and samples from metastatic lesions. Red dotted line indicates a cut-off for more or less advanced disease (above or below respectively, see Fig. 4). **p < 0.01. Pat: patient.

Cells were isolated from 83 tumour tissues obtained from 52 patients (52 primary tumours and 31 omental metastases) (Fig. 2b). We included both patients with biochemical and radiological suspicion of EOC undergoing primary debulking surgery and patients with biopsy-proven epithelial cancer of presumed tubo-ovarian origin. The latter group was treated with neoadjuvant paclitaxel and carboplatin therapy (Fig. 2b). Of the 83 samples, 37 (45%) yielded the number of cells required for establishing the desired number of tumour-bearing zebrafish embryos (i.e., 20 embryos from each tumour). However, 10 of these 37 samples were subsequently excluded due to non-EOC malignancy, analysis failure, or premature death of larvae (Fig. 2b). Hence, 27 samples (19 primary tumour and 8 metastatic lesions) from 23 patients were analyzed further. The characteristics of these 23 patients are presented in Table 1 and Supplemental Table 1. The most common histological subtype was high-grade serous carcinoma, and 70% of the patients had stage III or IV disease. Large differences in survival and growth were observed following the implantation of cells in zebrafish embryos (Fig. 3a–c). The tumours from one patient grew to more than twice the initial size, but most tumours exhibited partial regression after implantation in the zebrafish embryos. Both proliferating and apoptotic cells could be observed in the implanted tumours (Supplemental Fig. S1), suggesting that growth or partial regression is based on the balance between these processes in each sample.

**Table 1:** Patient characteristics

Category		Value (% or range)
Number of patients		23
Samples used		27
Samples from primary tumour		19 (70.4%)
Samples from metastases		8 (29.6%)
Mean age, years ± SD		63.7 ± 11.6 (43–82)
BMI, kg/m^2^ ± SD		26.1 ± 5.1 (16.5–37.5)
*Treatment*		
Primary surgery + adjuvant chemotherapy		13 (56.5%)
Outcome of surgery	Radical	11 (84.6%)
	Minimal residual tumour	0 (0.0%)
	Bulky residual tumour	2 (15.4%)
*NACT + Surgery*		6 (26.1%)
Outcome of surgery	Radical	4 (66.7%)
	Minimal residual tumour	0 (0.0%)
	Bulky residual tumour	2 (33.3%)
Diagnostic surgery + adjuvant chemotherapy		2 (8.7%)
Outcome of surgery	Radical	0 (0.0%)
	Minimal residual tumour	0 (0.0%)
	Bulky residual tumour	2 (100.0%)
*Only surgery*		2 (8.7%)
Outcome of surgery	Radical	2 (0.0%)
	Minimal residual tumour	0 (0.0%)
	Bulky residual tumour	0 (0.0%)
*FIGO Stage*		
Stage I–II		5 (21.7%)
Stage IIIA–C		11 (43.5%)
Stage IVA–B		5 (21.7%)
Stage X		2 (13.0%)

### Tumour dissemination in zebrafish embryos is associated with PFS and tumour stage

ZTX models could be established from paired primary tumours and metastatic lesions from four patients. The cells derived from the metastatic lesion of one patient grew significantly more compared to cells from the primary site, but this was not the case for the other three matched pairs (Fig. 3d). Metastatic dissemination of cells derived from metastatic lesions was significantly increased in two of four models compared to cells derived from the primary site (Fig. 3e).

**Fig. 4:**
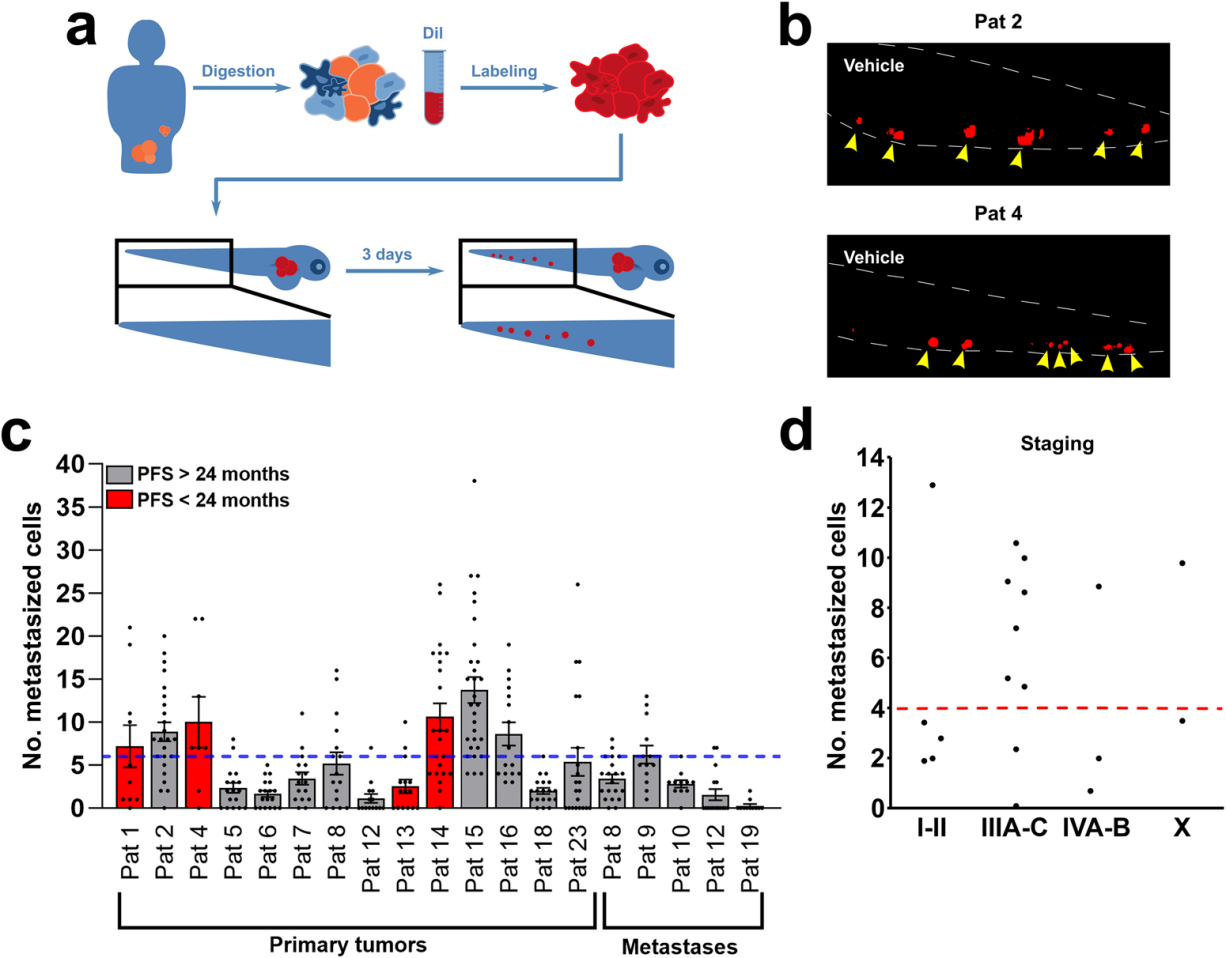
Dissemination of tumour cells as a tool for prediction of outcome. **a** Illustration of digestion, staining, and imaging of disseminated cells to caudal venous plexus (the area in the black box) of the zebrafish larvae. **b** Representative images of dissemination in the zebrafish larvae with tumour material from primary tumours of Patient 2 and Patient 4. Yellow arrowheads point to metastasized tumour cells (red). **c** Quantification of number of disseminated cells in the zebrafish larvae from primary tumours (*n* = 14, experiment done once) and metastatic lesions (*n* = 5, experiment done once) compared to patients who had a progression-free survival (PFS) of less than 24 months (red bars) with a cut-off value of 6 (blue dotted line). **d** Quantification of number of disseminated cells in the zebrafish larvae from primary tumours compared to staging with a cut-off value of 4 (red dotted line). *n* = 5, *n* = 9, *n* = 3, *n* = 2 for Stage I–II, Stage III A–C, Stage IV A–B, and Stage X (an initially unknown stage that was later confirmed to be Stage IV), respectively. All experiments were done once. Pat: patient.

Tumour cells injected from 17 patients could be evaluated for metastatic dissemination (Fig. 2b, Supplemental Fig. S2 and Fig. 4a–d). ZTX models established from patients that progressed within 24 months from completed treatment with Carboplatin + Paclitaxel generally showed higher dissemination compared to models generated from patients with >24 PFS. At a cut-off value of 6 disseminated cells, we found that 7 of 8 (88%) primary tumour ZTX models with a dissemination of less than 6 cells, i.e. models generated from patients 5, 6, 7, 8, 12, 13, 18 and 23, were generated from patients with >24 months PFS (grey bars in Fig. 4c). Conversely, 3 of 4 models (75%) generated from primary tumour samples from patients with <24 months PFS (red bars in Fig. 4c) exhibited above cut-off metastatic dissemination.

Five patients were diagnosed with FIGO stage I or II (disease limited to the ovaries and/or the fallopian tubes and/or spread (only) to adjacent tissues). Most patients with EOC, however, are diagnosed with advanced stage disease; in our material, nine patients were diagnosed with FIGO stage III disease, which is characterized by metastases to lymph nodes and/or abdominal spread, and five patients with FIGO stage IV disease, which is characterized by distant metastases. Interestingly, ZTX dissemination below 4 cells per embryo was found for 4 of 5 (80%) patients diagnosed with stage I or II disease, whereas 7 of 9 (78%) of the ZTX models established from patients with stage III disease exhibited dissemination above the 4-cell cut-off (Fig. 4d). ZTX models established from patients with stage IV disease exhibited dissemination below the 4-cell cut-off in 3 of 5 cases. Taken together, below cut-off dissemination in the ZTX models is associated with stage I/II disease, and above cut-off dissemination in the ZTX models is associated with stage III disease.

### Treatment of ZTX models with carboplatin or paclitaxel

**Fig. 5:**
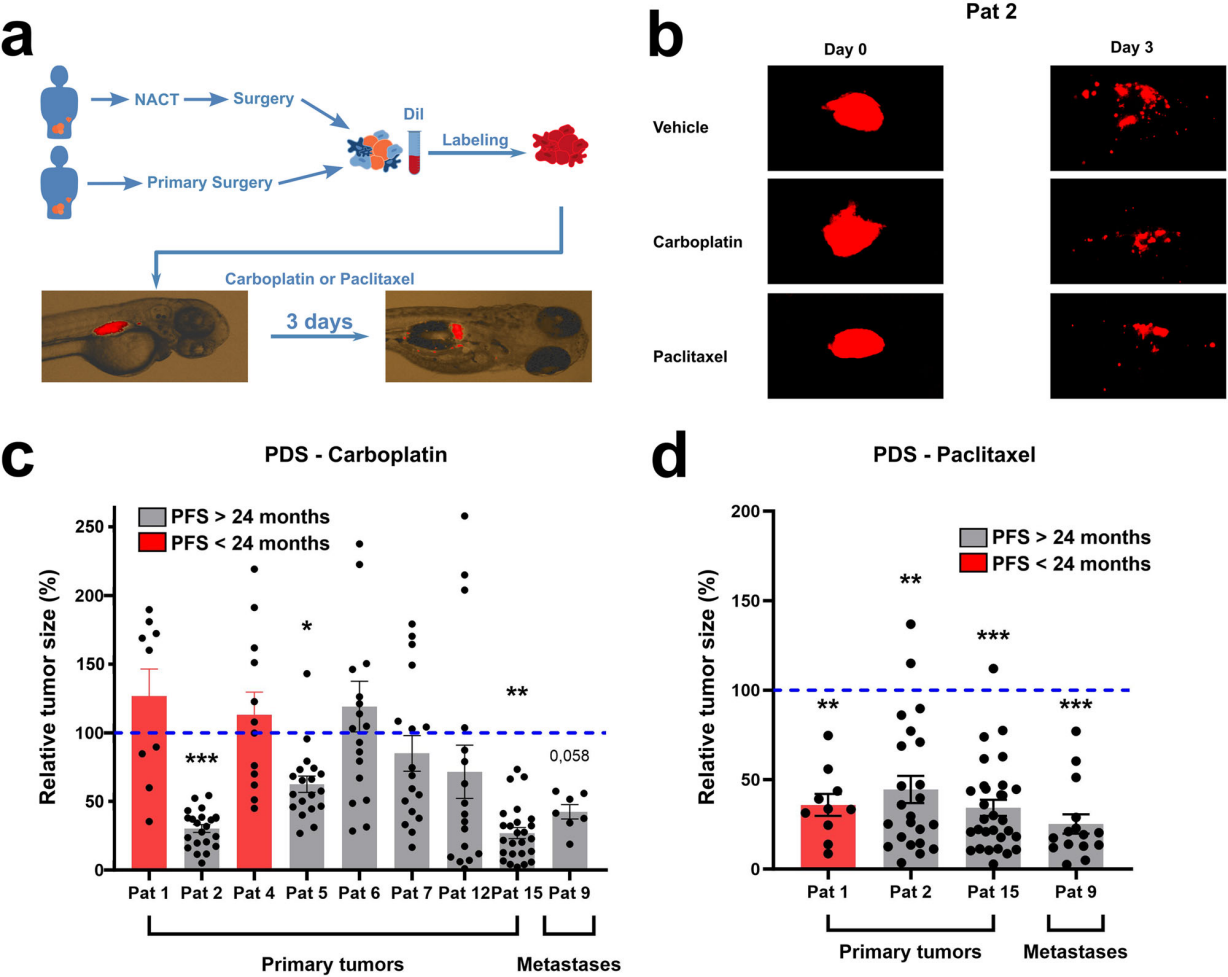
Treatment outcome in ZTX models is associated with PFS in the corresponding patients. **a** Illustration of tumour samples being labelled and injected into the zebrafish larvae. The patients had either been treated with neoadjuvant cytostatic therapy (NACT) before surgery or treated with primary surgery. **b** Representative images of change in tumour size between day zero and three compared between vehicle group and either carboplatin (10 μg/mL) or paclitaxel (20 μg/mL) in Patient 2. **c**, **d** Comparison of change in tumour size in the zebrafish larvae between vehicle group and groups treated with either carboplatin (10 μg/mL) (**c**) or paclitaxel (20 μg/mL) in samples from either primary tumours or from metastatic lesions in patients treated with primary debulking surgery (PDS). Patients with a progression-free survival (PFS) of less than 24 months are indicated with a red bar. Control tumour size (100%) is indicated by a blue dashed line. All experiments were done once *p < 0.05, **p < 0.01, ***p < 0.001.

Carboplatin or paclitaxel was administered in the water after implantation of patient tumours in zebrafish larvae (Fig. 5a) using a smaller exploratory cohort of ZTX models derived from the tumours of 13 patients that had completed adjuvant treatment with Carboplatin + Paclitaxel combination therapy, and for whom data on their PFS was available (see Fig. 2b). The tumours from these patients were selected because they did not regress beyond 80% in the non-treated control group, and therefore retained a sufficient size to allow evaluation of an additional drug-induced anti-cancer effect. Of the nine tumours exposed to carboplatin, the tumour volumes of four were found to be significantly or near-significantly (*p* < 0.06) reduced, but five tumours did not exhibit any carboplatin-induced growth retardation (Fig. 5b, c). All four ZTX models showing carboplatin-induced growth retardation were established from patients having >24 months PFS. Conversely, ZTX models established from the two patients that progressed in this cohort were in both cases not growth retarded by carboplatin. Only four ZTX models were exposed to paclitaxel due to our concerns about relevant dosing. The growth of all four tumours exposed to paclitaxel was retarded (Fig. 5d).

**Fig. 6:**
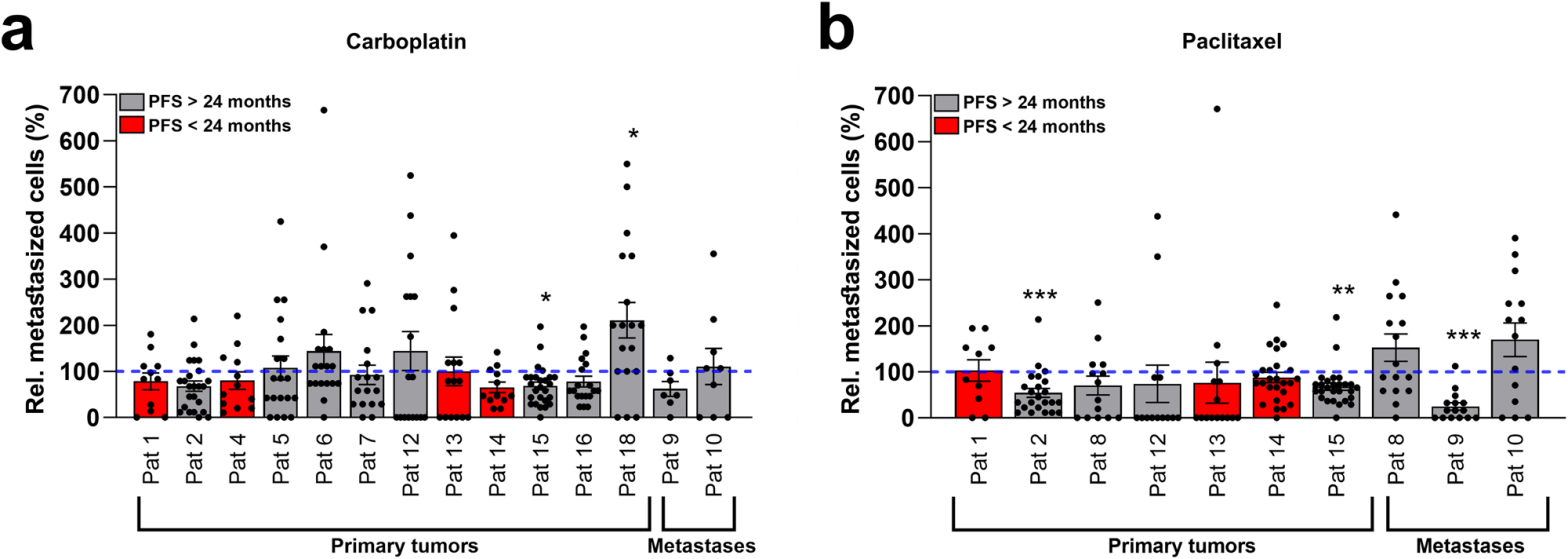
Carboplatin and paclitaxel do not inhibit metastasis in patient-derived ZTX models of EOC. **a**, **b** Comparison of changes in average number of disseminated cells (rel. number of metastasized cells) between vehicle and treatment with either carboplatin (10 μg/mL) (**a**) and paclitaxel (20 μg/mL) (**b**) in samples from either primary tumours or from metastatic lesions. Patients with a progression-free survival (PFS) of less than 24 months are indicated with a red bar. Dissemination in non-treated controls (100%) is indicated by the blue dashed line. All experiments were done once. *p < 0.05, **p < 0.01, ***p < 0.001. Pat: patient.

We next investigated how tumour dissemination in the embryos was affected by exposure to carboplatin or paclitaxel. Treatment with carboplatin or paclitaxel only significantly reduced metastatic dissemination in 1 of 14 and 3 of 10 of the evaluated patients, respectively (Fig. 6a, b).

## Discussion

Procedures to aid treatment decisions in oncology need to be reasonably swift. The median time between diagnosis and initiation of therapy is in the order of one to three months for malignant diseases such as colon cancer and prostate cancer^29^. Mouse PDX models have been developed but are of limited clinical use due to the time (about six months) that is required for the establishment of the model and for drug testing^30,31^. In contrast, the zebrafish PDX model is simple, rapid, and cost-effective and therefore offers distinct advantages^32^.

Here, we demonstrate the proof-of-concept for a 3-day ovarian cancer ZTX assay that tests drug sensitivity. Ovarian cancer tissues are notoriously difficult sources for generating PDX models in mice, having a success rate of a mere 10–20%^33^. However, our assay had an implantation and engraftment success rate of 45% and 70%, respectively, meaning that large numbers of tumour-baring zebrafish larvae could be generated for almost one-third of the patients. Furthermore, here we address the problem of developing a procedure that overcomes the logistic hurdles in sample handling. Debulking ovarian cancer surgery typically lasts several hours, so resected tumours are often only available late during a working day. Moreover, it takes several hours to isolate tumour cells and inject them into 20 embryos per treatment group (up to 100 embryos). The procedure used here—i.e., cryopreserving tumour pieces in DMSO—offers the possibility of better experimental planning and better use of resources. We found that the success rate of successful tumour implantation was comparable between frozen and fresh tumours. Rather than using tissue samples to isolate EOC cells for the ZTX models, ascites fluid could probably be used as it is often rich in tumour cells^34^. This tactic would facilitate the cell isolation process and possibly lead to ZTX models being established from a larger proportion of the patients.

Many studies have indicated that ZTX models can be used to predict the response of solid tumours to various therapies^35^. For example, a gastric cancer study^19^ reported the successful injection of cells from 28 patients (from a total of 46). Of these patients, 27 received postoperative adjuvant chemotherapy. Drug sensitivity in the ZTX models was inversely associated with recurrence 6–12 months after surgery. Fior et al.^18^ generated ZTX models from five colorectal tumours and exposed these to FOLFOX (folinic acid, fluorouracil, and oxaliplatin). Responses to the ZTX assay were inversely correlated to relapse after surgery. In a study of 24 patients with pancreatic, colon, or gastric cancers, Usai et al. reported encouraging results with regard to correlations between drug sensitivities in the ZTX assay and clinical responses^20^. Di Franco described an effective procedure for establishing pancreas cancer ZTX models and reported responses to various chemotherapies but did not attempt to correlate these responses to clinical responses^36^. Hua et al. established 13 ZTX models from 21 patients diagnosed with NSCLC and evaluated treatment modalities involving tyrosine kinase inhibitors, pemetrexed/platinum, or docetaxel/platinum (DP)^37^. Although the number of patients Hua et al.’s study was limited (*n* = 13), response was evaluated using both imaging methods and serum tumour markers: concordant results between clinical responses and those obtained from ZTX models were obtained for 10/13 patients/tumours. Our results using ovarian cancer ZTX models are also worth further examination: 2 of the 9 patients evaluated for sensitivity to carboplatin progressed within the 2-year follow-up. Both of these patients were included among the five ZTX models that showed poor responses to carboplatin. If this result holds in larger studies, the ZTX models will have excellent sensitivity (100% in this small cohort) for predicting non-responders. Furthermore, 7 of the 9 patients did not progress within the 2-year follow-up. The four ZTX models that showed response to carboplatin were all included among those generated from these 7 stable remission patients. If this result should also hold in larger studies in the future, the ZTX models will also have excellent sensitivity (again 100% in our small cohort) for predicting patients that gain clinically meaningful survival benefits from (in our case) carboplatin-containing treatments. Correlations between short-term drug sensitivity and long-term clinical outcomes are, however, not expected to be absolute as clinical drug resistance can be explained by tumour heterogeneity, regrowth of “cancer stem cells” and other factors that are not evaluated during the 3-day ZTX assay. Similarly, clinical drug response among those showing resistance in the ZTX models could be explained by a potentially complete removal of the tumour during debulking surgery, the ability of the patient’s immune system to clear remaining tumour cells post-surgery, potentially augmented efficacy from the clinical formulation, of other drugs used in combination, drug-drug interactions, or other factors that were not evaluated in the ZTX models but that could interact with the treatment or work independently to improve survival in the patient. In short, the model has to be further tested and evaluated to see if it is in treatment prediction or disease prognostication it has the highest potential usefulness. However, a model to identify tumours likely resistant to therapy would provide a tool for better clinical management of patients.

The zebrafish model provides a versatile tool in drug research and has been used for large-scale drug screening projects^38^. There are, however, many outstanding questions with regard to pharmacology. As the skin of zebrafish larvae is permeable to drugs, they can be administered to the embryo medium easily^39^. Although phase I and phase II metabolism is generally conserved between zebrafish and mammals^40^, it is unclear how drugs distribute in zebrafish embryos after uptake through the skin and the extent they are exposed to detoxifying enzymes. We found that paclitaxel was not toxic to developing embryos even at doses at the extremely high concentration of 100 μg/mL (~120 μM). For comparison, the IC_50_ for this drug on proliferating human fibroblasts is ~35 nM^41^. The finding that developing embryos, which contain rapidly proliferating cells, are insensitive to paclitaxel is likely due to poor solubility of the drug in water. Paclitaxel is commonly dissolved in a formulation containing Chremophor mixed 1:1 with ethanol and is bound to plasma proteins by >90% after administration^42^. As these conditions cannot be mimicked in aquatic zebrafish embryo cultures, there is a limitation with regard to exposure. We found dose-dependent effects of paclitaxel on the growth of transplanted IGROV-1 cells and on the growth of three patient-derived ZDX models at concentrations above the limit of solubility. This finding could be explained by a dynamic flow from the insoluble drug to the water phase and subsequent accumulation in embryos. As paclitaxel dissolves slowly in water^43^, higher doses could lead to more rapid mobilization from the solid phase into water. Although the zebrafish model is very convenient for pharmacological studies, some caution should be taken during the analysis of drugs showing poor solubility. Evaluating the anti-tumour efficacy in zebrafish tumour models by adding Paclitaxel to the embryo water is, however, an approach that has been previously validated by others^44,45^. These prior studies used Paclitaxel at doses close to or exceeding 1 μM, suggesting that applying Paclitaxel to the water at >10X of the IC50 dose could be appropriate in spite of the currently unexplained potential pharmacological issues associated with this approach.

ZTX models have the potential to be used for drug response evaluation of EOC, but the models need to be refined and tested on larger cohorts and possibly using ascites fluid rather than tumour tissue. The rapid assessment of clinical tumours for drug sensitivity is an interesting possibility. A complete correlation between drug sensitivity in a short-term assay and clinical progress over several months is not expected. However, the identification of tumours that do not respond to specific treatment modalities leads to the possibility to choose other options. We believe that the possibility is sufficiently interesting to warrant further development of the technical aspects of the ZTX model pipeline to increase the rate of success of the procedures and to provide actionable data for a larger proportion of EOC patients.

In conclusion, here we show that ZTX models can be established from surgical EOC biopsies both from primary and metastatic sites. In addition, in a small exploratory study, we found that the response to carboplatin as well as metastatic dissemination of tumour cells within the zebrafish larvae mirror durable treatment outcome and tumour stage, respectively, in the patients. Therefore, ZTX models might become a promising tool for identifying patient populations that are likely to gain durable remission from carboplatin as well as those at risk of developing resistance in the short term after treatment onset. That is, these models could help explain the prognosis of the patients following a specific treatment protocol and therefore help healthcare providers individualize treatment plans. This knowledge could improve the chances that an alternative treatment with a durable therapeutic outcome is quickly identified, reducing the risk of disease relapse for a larger proportion of EOC patients.

## Methods

### Patient recruitment and ethics

Women who were referred to the department of Obstetrics and Gynecology at the University Hospital in Linköping for exploratory laparotomy due to presumed or verified advanced EOC were asked to participate in the study. After written informed consent, tumour tissue was resected at the laparotomy and placed in a 50 mL falcon tube at room temperature.

The sample was collected within 2 h and processed for cryopreservation. The study was run in full compliance with the declaration of Helsinki and was approved by the Ethical Board at Linköping University (Dnr. 2017/358-31; date of approval: August 16, 2017) prior to the recruitment of the first patient.

Information on disease characteristics, including histopathological subtype, Federation of Gynecology and Obstetrics (FIGO) stage, surgical and oncological outcome, and survival status were retrospectively collected from the medical files.

### Reagents

NaCl (Cat #S5886), CaCl_2_ (Cat #C8106) and dimethyl sulfoxide (DMSO, #276855-100) were purchased from Sigma. Low-yield tissue-disruptor Mix (Cat #P01-3002) and Cell Implantation Resuspension Medium (#P03-2001) were purchased from BioReperia, and RPMI-1640 culture medium (Cat #LM-R1637/500) was purchased from Biosera. Foetal bovine serum (Cat #97068-085), MgSO4 (Cat #0662), KCl (Cat #26764.232), and phosphate buffered saline (PBS, #E403-500) were purchased from VWR. Gentle MACS™ C-tube (Cat #130096334) was purchased from Miltenyi Biotec. Trypan blue 0.4% solution (Cat #15250061) and Fast-DiI™ oil (#D3899) were purchased from ThermoFisher Scientific. Carboplatin (Cat #S1215) and paclitaxel (Cat #S1150) were purchased from Selleckchem. 1-Phenyl-2-Thiourea (aka PTU, Cat #L06690) was purchased from Alfa Aesar. Penicillin-Streptomycin (#L0022-100) and Trypsin/EDTA (#MS0158100U) were purchased from Biowest.

### Cryopreservation of tumour tissues

Tumour tissues were cut into smaller pieces (maximum of 2 × 2 × 2 mm) using sterilized forceps and scalpel blades. A maximum of 0.5 g of tissue was added to 1.5-mL cryotubes containing 1 mL of cryopreservation medium (90% FCS, 10% DMSO) and immediately transferred to a CoolCell® freezing container at −80 degrees. Cryopreserved samples were transported on dry ice to BioReperia.

### Zebrafish strains, maintenance, and ethics

Adult zebrafish were maintained at the zebrafish facility at Linköping University (Linköping, Sweden) and maintained under standard housing conditions as previously described^46^. Tg(fli1a:EGFP)^y1^ fish^47^ were purchased from ZIRC (Oregon). Zebrafish embryos were produced by natural breeding and maintained in E3 embryo medium, which contained 0.286 g NaCl, 0.048 g CaCl_2_, 0.081 g MgSO_4_, and 0.0126 g KCl per litre at pH 7.2 and supplemented with 0.2 mM PTU (E3/PTU) at 28.5 °C prior to cell implantation. All experiments were concluded before the development of autonomous feeding behaviour at approximately the 5-dpf stage and did therefore not require ethically approved animal protocols.

### Implantation of PDX models into zebrafish larvae and drug treatment

Fresh tumour tissue samples were cut into small pieces or thawed in the case of cryopreserved samples, and washed gently by inversion with 10 mL of RPMI-1640 medium supplemented with 10% FBS (RPMI-FBS). Next, 5 mL of low-yield tissue-disruptor mix were added and the tissue suspension was transferred to a Gentle MACS™ C-tube and dissociated in a gentleMACS™ Octo Dissociator for 30 min at 37 °C. The resulting single-cell suspension was washed with RPMI-FBS, filtered through a 30 μm cell strainer to remove undigested tissue pieces, and labelled with 8 μg/mL of Fast-DiI™ oil (ThermoFisher Scientific Cat #D3899) in RPMI supplemented with 2% FBS for 30 minutes at 37 °C. Cells were then washed and re-filtered, and cell viability and count were determined using the trypan blue-exclusion method.

DiI-labelled cells were resuspended in a cell implantation resuspension medium, and approximately 500 cells were implanted subcutaneously in the dorsal perivitelline space for 2-day-old zebrafish embryos as previously described^48^. 2-day old embryos were chosen as the perivitelline space was accessible for implantation and the yolk was sufficiently mature to reduce the risk of damage during the implantation process. Embryos in which cells were erroneously injected into the yolk or bloodstream were excluded from further analysis. Implantation success was defined as the proportion of patient tissues from which enough cells could be retrieved, labelled and implanted in at least 20 embryos. Successfully implanted embryos were identified and selected under a fluorescent stereoscope model M205 FA (Leica Microsystems CMS GmbH). Primary tumours were imaged using the Leica Application Suite X (LAS X) software v3.7.1.21655 (Leica Microsystems CMS GmbH), and the embryos were randomly stratified into control or treatment groups of 20 embryos per group and incubated for 72 hours at 36 °C in E3/PTU water containing carboplatin (10 μg/mL), paclitaxel (20 μg/mL), both carboplatin (10 μg/mL) and paclitaxel (20 μg/mL), or vehicle. 36 °C was chosen as this was the temperature closest to the preferred temperature of the human cells that the embryos could tolerate. The embryos were then re-imaged using the LAS X software, and images of the primary tumour implantation site and the main metastatic site in the caudal venous plexus were obtained.

### Analysis of tumour growth or regression, and dissemination in zebrafish larvae

Images were acquired at 2048 × 2048 pixels resolution at an image magnification of 100x with no binning, giving a pixel size of 6.5 × 6.5 μm. Images obtained within 2 h from implantation (0 days post injection, dpi) and after 72 hours of incubation (3 dpi) were obtained and analyzed using the HuginMunin software v2.7.0.0 (BioReperia AB, Linköping, Sweden). Tumour size refers to the area tumour cell-specific fluorescent pixels at 0 dpi and 3 dpi. The relative tumour size was calculated by dividing the tumour area at 3 dpi by the area at 0 dpi in the same embryo. This value was multiplied by 100 to convert to a percentage. A value lower than 20% (indicative of >80% spontaneous regression) in the vehicle-treated control group was regarded as implantation failure, and such samples were excluded from further analysis. Metastatic dissemination was evaluated by counting the number of disseminated cell-sized DiI-positive signals in the images of the caudal venous plexus.

### Drug toxicity assay in zebrafish

Zebrafish embryos were collected and maintained in E3/PTU at 28.5 °C for 48 h. Two days after fertilization, the embryos were randomly separated into treatment groups of 20 embryos per group. Treatment with either 5, 10, 40, 80, and 100 μg/mL of carboplatin or 5, 10, 40, 80, and 100 μg/mL paclitaxel was done by adding the drugs to the E3/PTU water. The embryos were incubated at 36 °C. The number of living embryos and parameters of non-lethal toxicity, including pericardial oedema, head and tail necrosis, bending or malformation of head and tail, teratogenesis, brain haemorrhage, and yolk sack oedema, were recorded 72 hours after drug exposure.

### Drug efficacy assay in zebrafish

IGROV-1 cells grown in DMEM medium were labelled with DiI and implanted in the dorsal perivitelline space of 2-day-old zebrafish embryos as previously described^49^. Tumours were imaged within 2 h of implantation, and tumour-bearing embryos were treated with either vehicle, 0.4, 2, and 10 μg/mL of carboplatin, or 0.4, 2, and 10 μg/mL of paclitaxel for 3 days when they were re-imaged. Changes in relative tumour size were evaluated as described for the EOC samples and compared between drug treatment and vehicle-control groups.

### Statistics

The ZTX models were performed in a blind fashion. As binominal distribution was confirmed using the Kolmogorov-Smirnov test, the data are shown as means ± SEM from representative experiments. Statistical comparisons between two groups were made using two-tailed, unpaired Student’s t-test assuming equal variance of the data sets. *N*-values represent the number of larvae analyzed within the group. All statistical analyses were performed and generated using Excel 2019 (Microsoft).

### Supplementary information

Supplemental Information

## Data Availability

All data generated or analyzed during this study are included in an aggregated form in this published article and its supplementary information files. The non-aggregated raw datasets are available from the corresponding author on reasonable request.

## Code availability

The software used for this study, including the applicable versions and commercial suppliers, is described in the Materials and Methods section.
