# Supplementary material for: Zebrafish tumour xenograft models: a prognostic approach to epithelial ovarian cancer
Source: NPJ Precis Oncol. 2024 Feb 27;8:53. doi: 10.1038/s41698-024-00550-9 (PMC10899227; doi:10.1038/s41698-024-00550-9)

Supplemental Information

Supplemental Table 1: Patients characteristics

Patient number	Age at diagnosis	Diagnosis	Histological subtype	Stage	BRCA-status	Treatment	Surgery	Outcome of surgery	PFS
1	72	ovarian cancer	high grade serous	IIIB	wild type	P/K	PDS	R0	16
2	70	ovarian cancer	seromucinous	IVB	wild type	P/K/B	PDS	R0	NA
3	59	abdominal cancer	endometrioid grade 3	X	wild type	K	diagnostic	R2	0
4	66	ovarian cancer	high grade serous	IIIC	wild type	P/K	PDS	R0	10
5	55	fallopian tube cancer	high grade serous	IIIC	BRCA1	P/K/Pa	PDS	R0	NA
6	58	fallopian tube cancer	high grade serous	IIB	wild type	PLD/K	PDS	R0	NA
7	82	ovarian borderline tumor	mucinous	IA	unknown	0	PDS	R0	NA
8	47	ovarian cancer	high grade serous	IIIC	BRCA1	LoF	NACT+DPDS	R0	LoF
9	74	fallopian tube cancer	high grade serous	IIIC	wild type	PLD/K	PDS	R0	27
10	73	fallopian tube cancer	high grade serous	IVB	BRCA1	P/K/B	NACT+DPDS	R0	NA
11	65	abdominal cancer	high grade serous	X	unknown	P/K	diagnostic	R2	0
12	44	ovarian cancer	high grade serous	IVB	wild type	P/K/B	PDS	R0	NA
13	63	ovarian cancer	high grade serous	IVB	wild type	P/K/B	PDS	R0	6
14	68	ovarian cancer	high grade serous	IIIC	wild type	P/K/B	NACT+DPDS	R0	18
15	55	ovarian cancer	high grade serous	IIA	wild type	P/K	PDS	R0	NA
16	48	ovarian cancer	endometrioid grade 3	IIIC	BRCA2	P/K/Pa	PDS	R0	NA
17	43	fallopian tube cancer	high grade serous	IIIA1	wild type	P/K/B	PDS	R2	NA
18	81	ovarian cancer	high grade serous	IA	wild type	P/K	PDS	R0	NA
19	61	ovarian cancer	carcinosarcoma	IVB	unknown	P/K	NACT+DPDS	R0	Lof
20	76	fallopian tube cancer	high grade serous	IIIC	wild type	P/K	PDS	R2	11
21	74	fallopian tube cancer	carcinosarcoma	IIIC	wild type	P/K/B	NACT+DPDS	R0	2
22	57	ovarian cancer	carcinosarcoma	IIIC	BRCA1	P/K/B	PDS	R2	9
23	75	ovarian cancer	endometrioid grade 2	IA	unknown	0	PDS	R0	41

P - paclitaxel K - carboplatin B - bevacizumab Pa - parpinhibitor PLD - pegylated liposomal doxorubici

LoF - lost to follow up NA - not applicable

PDS - primary debulking surgery NACT+DPDS - neoadjuvant chemotherapy and delayed primary debı
surgery

R0 - macroscopic radical R2 - bulky disease

Supplemental Figure S1

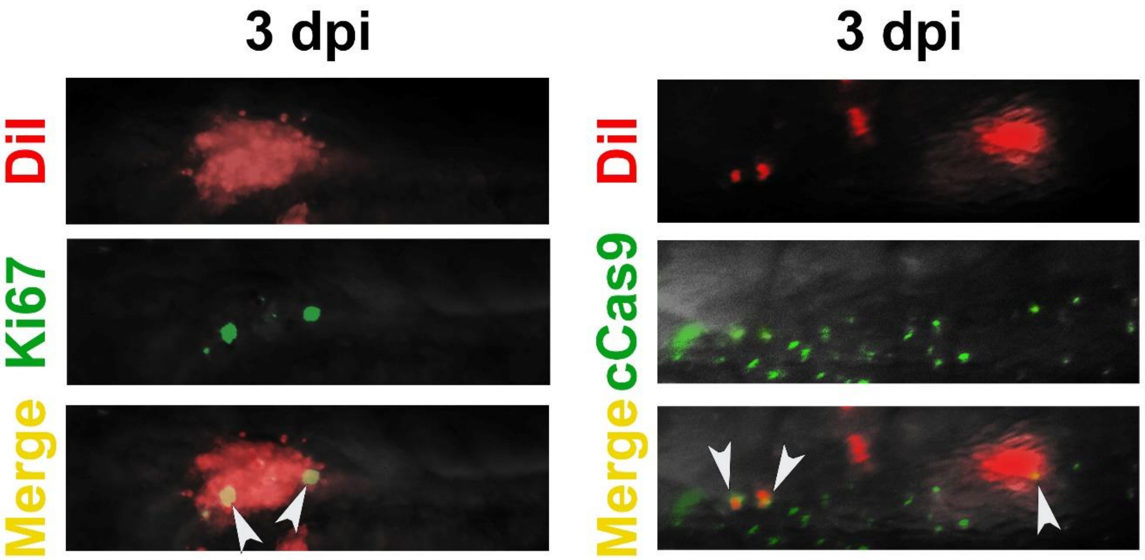

Supplemental Figure S2

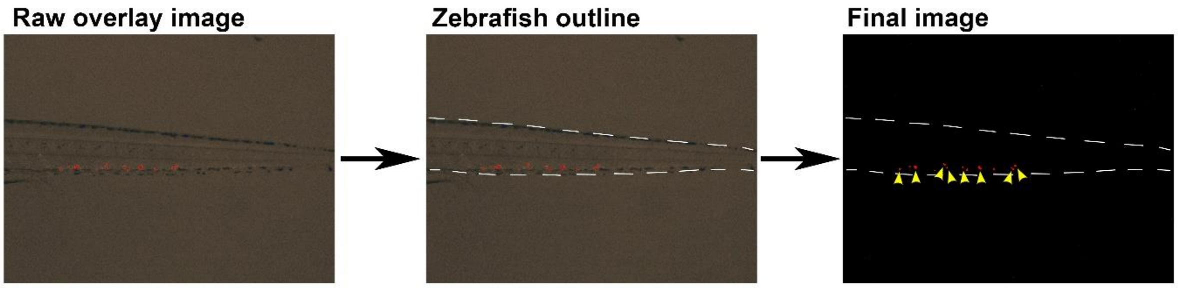

Supplement: Supplementary file 1 — Supplemental Information [file 41698_2024_550_MOESM1_ESM.pdf]
